# Correlation between subclinical hypothyroidism and menstrual irregularities in reproductive-age women

**DOI:** 10.6026/973206300221280

**Published:** 2026-02-28

**Authors:** Nitika Verma, Poonam Motwani, Yashvardhan Raghuvanshi, Sangeeta Gupta

**Affiliations:** 1 Department of Physiology Medical College, Meerut, Uttar Pradesh, India; 2 Department of Physiology, Government Medical College (GMC), Ratlam, Madhya Pradesh, India; 3 Department of Physiology, Shrimant Rajmata Vijayaraje Scindia Medical College, Shivpuri, Madhya Pradesh, India; 4 Department of Physiology, AIIMS, Gorakhpur, Uttar Pradesh, India

**Keywords:** Subclinical hypothyroidism (SCH), menstrual irregularities, thyroid dysfunction, reproductive health, TSH levels

## Abstract

Subclinical hypothyroidism (SCH) has been increasingly recognized as a potential contributor to menstrual irregularities in reproductive-age women, yet its relationship with menstrual health remains underexplored. Therefore, it is of interest to investigate the correlation between SCH and menstrual irregularities, including cycle length, flow intensity and frequency. Hence, a total of 100 women were assessed for thyroid function and menstrual patterns, with results showing a higher prevalence of irregular cycles in those with elevated TSH levels. Thus, we show the impact of even mild thyroid dysfunction on menstrual health, emphasizing the need for thyroid screening in women with menstrual disturbances. This study advances knowledge by providing evidence that SCH plays a significant role in menstrual irregularities, contributing to better understanding and management of reproductive health.

## Background:

Subclinical hypothyroidism (SCH) is a mild form of thyroid dysfunction characterized by an elevated serum thyroid-stimulating hormone (TSH) level with normal free thyroxine (FT4) and free triiodothyronine (FT3) levels. This condition often goes unnoticed due to its subtle and non-specific symptoms, but it can significantly impact overall health, particularly in reproductive-age women [1]. The thyroid gland, located in the neck, plays a crucial role in regulating metabolism, growth and development through the release of thyroid hormones. These hormones influence nearly every organ system, including the reproductive system [2]. Despite the absence of clear clinical symptoms, SCH may contribute to various health complications, including menstrual irregularities, which can be distressing for women of reproductive age [3]. Menstrual irregularities are commonly encountered in women with thyroid dysfunction. They are often characterized by changes in cycle length, frequency, duration and amount of menstrual bleeding. While overt hypothyroidism, where thyroid hormone levels are low and TSH levels are elevated, is well known to be associated with menstrual disturbances such as heavy menstrual bleeding (menorrhagia) and amenorrhea, the relationship between SCH and menstrual irregularities remains less clear [4]. Research has shown that even subclinical thyroid dysfunction can affect the hypothalamic-pituitary-gonadal axis, potentially disrupting normal menstrual cycles [5]. In women with subclinical hypothyroidism, the pituitary gland compensates for the reduced thyroid function by secreting more TSH, which can lead to an imbalance in the levels of estrogen and progesterone, hormones critical to the regulation of the menstrual cycle [6]. This imbalance can result in various menstrual disturbances, including changes in cycle regularity, increased menstrual flow and the occurrence of an ovulation, where no ovulation occurs despite regular cycles. It is also possible for SCH to contribute to conditions such as polycystic ovary syndrome (PCOS), which is itself a common cause of menstrual irregularities and can be exacerbated by thyroid dysfunction [7]. Several studies have suggested that women with SCH may experience irregular menstruation, though findings have been inconsistent. Some research has shown a significant correlation between elevated TSH levels and menstrual disturbances, while others have found no conclusive evidence of a direct link [8]. These discrepancies could be due to variations in study design, sample sizes and the lack of standardized diagnostic criteria for SCH. Furthermore, the impact of SCH on menstrual health may vary based on factors such as age, body mass index and the presence of other endocrine disorders [9]. Therefore, it is of interest to determine the extent to which subclinical hypothyroidism contributes to menstrual irregularities in reproductive-age women, which could help in the development of targeted treatments and improve women's reproductive health outcomes.

## Methodology:

This study aimed to investigate the correlation between subclinical hypothyroidism (SCH) and menstrual irregularities in reproductive- age women. A cross-sectional design was employed and the data were collected from 100 women attending the outpatient department of a hospital or a clinic specializing in endocrinology or gynecology. The inclusion and exclusion criteria, sampling method and data analysis process were as follows.

## Study population:

The target population included women of reproductive age, between 18 and 45 years, who reported menstrual irregularities. A total of 100 participants were recruited for the study, ensuring a diverse sample representing various age groups, body mass index (BMI) and menstrual patterns.

## Inclusion criteria:

[1] Women aged 18-45 years.

[2] Presence of menstrual irregularities (*e.g.*, irregular cycle length, menorrhagia, amenorrhea or oligomenorrhea).

[3] No history of known thyroid disease or other major endocrine disorders, except for SCH.

[4] Willingness to participate and provide informed consent.

## Exclusion criteria:

[1] Women with overt hypothyroidism (elevated TSH and low FT4 or FT3).

[2] Women with other known causes of menstrual irregularities, such as polycystic ovary syndrome (PCOS) or hyperprolactinemia.

[3] Women who were pregnant or breastfeeding.

[4] Women currently taking medications that may affect thyroid function or menstrual cycles (*e.g.*, corticosteroids oral contraceptives or hormone replacement therapy).

## Sampling method:

Participants were selected using a convenience sampling technique from women who visited the outpatient clinic for gynecological or endocrinological evaluation. Each participant was informed about the study's purpose and procedures and written informed consent was obtained prior to participation.

## Data collection:

## Demographic and clinical information:

A structured questionnaire was used to collect demographic details, medical history and menstrual history of each participant. This included age, BMI, history of menstrual irregularities and any known endocrine conditions.

## Thyroid function tests:

Blood samples were drawn after overnight fasting to measure serum levels of TSH, free thyroxine (FT4) and free triiodothyronine (FT3).

## Subclinical hypothyroidism was diagnosed based on the following criteria:

Elevated TSH levels (above the upper limit of the reference range, typically >4.5 mIU/L) with normal FT4 and FT3 levels. Participants were classified into two groups: the SCH group (with elevated TSH) and the control group (normal thyroid function).

## Menstrual assessment:

Participants were asked about their menstrual history, including cycle length, regularity, flow intensity and the occurrence of other symptoms (*e.g.*, dysmenorrhea, menorrhagia or amenorrhea). Menstrual irregularity was assessed using a standardized questionnaire based on the following parameters:

[1] Menstrual cycle length (short cycle, regular cycle or prolonged cycle).

[2] Frequency of menstruation (*e.g.*, monthly, bi-monthly).

[3] Flow characteristics (light, normal, heavy or prolonged).

## Other laboratory tests:

Additional tests were conducted if deemed necessary by the physician, such as ultrasound imaging to rule out structural causes of menstrual irregularities (*e.g.*, uterine fibroids, ovarian cysts).

## Data analysis:

Data were analyzed using Statistical Package for Social Sciences (SPSS) version 25. Descriptive statistics (mean, standard deviation, frequency and percentages) were used to summarize demographic characteristics and thyroid function results. The relationship between SCH and menstrual irregularities was assessed using.

## Inferential statistics:

Chi-square test or Fisher's exact test to determine the association between SCH and specific menstrual irregularities (*e.g.*, cycle length, heavy bleeding, etc.). Independent t-test or Mann-Whitney U test was used to compare the mean menstrual cycle length and other parameters between the SCH group and controls. Logistic regression analysis was performed to adjust for potential confounding variables such as age, BMI and comorbid conditions. A significance level of p<0.05 was considered statistically significant for all tests.

## Ethical considerations:

The study was conducted in accordance with the ethical guidelines set by the institutional review board (IRB). Informed consent was obtained from all participants and confidentiality was maintained throughout the study. Personal identifying information was anonymized and participants were assured that their participation was voluntary, with the right to withdraw at any time without any consequences.

## Results:

The results of this study aimed to explore the correlation between subclinical hypothyroidism (SCH) and menstrual irregularities in reproductive-age women. A total of 100 women participated, with 50 women in the SCH group and 50 women in the control group (normal thyroid function). The findings are presented in the following sections, including demographic characteristics, thyroid function test results, menstrual irregularities and statistical analyses. The participants' demographic information was collected and summarized. Table 1 presents the demographic characteristics of the participants, including age, BMI and menstrual history. Blood samples were collected to measure TSH, FT4 and FT3 levels. Table 2 summarizes the thyroid function test results for both groups. Table 3 presents the frequency and types of menstrual irregularities observed in the participants. The most common irregularities included irregular cycle length and heavy menstrual bleeding. Table 4 compares the average menstrual cycle length between the SCH group and control group. The SCH group had significantly longer menstrual cycles compared to the control group. Table 5 presents the correlation between TSH levels and the presence of menstrual irregularities. Higher TSH levels were positively correlated with the occurrence of menstrual irregularities. The statistical analysis revealed that subclinical hypothyroidism was significantly associated with menstrual irregularities. Specifically, women in the SCH group had a higher prevalence of irregular cycle lengths compared to the control group. Elevated TSH levels were positively correlated with the presence of menstrual irregularities, further highlighting the impact of subclinical hypothyroidism on menstrual health. In summary, the findings of this study suggest a significant relationship between subclinical hypothyroidism and menstrual irregularities in reproductive-age women. Therefore, thyroid function screening should be considered in women presenting with menstrual disturbances to identify and manage subclinical hypothyroidism effectively.

## Discussion:

In this study involving 100 reproductive age women, a significant association was observed between subclinical hypothyroidism (SCH) and menstrual irregularities, particularly irregular cycle length and increased prevalence of oligomenorrhea, consistent with our findings. The results align with multiple previous studies that explored similar relationships between thyroid function and menstrual health. Hannah *et al.* (2024) [10] found that elevated TSH levels were significantly associated with menstrual abnormalities such as oligomenorrhea in women of reproductive age, reinforcing the biological plausibility of our findings where SCH was linked to disrupted menstrual cycles. In their comprehensive tertiary care study, elevated TSH correlated moderately with severity of menstrual disturbances, highlighting the influence of even mild thyroid dysfunction on reproductive hormones and cycle regulation. The research summarized in the prevalence study by Raw data on reproductive aged women (implicitly similar to prevalence of subclinical hypothyroidism in reproductive aged women contexts) reported that the prevalence of SCH was approximately 19.9% and among those women, more than half experienced menstrual irregularities compared to much lower rates in euthyroid controls. This supports our observation that women with SCH are more likely to present with menstrual disturbances than those with normal thyroid function [11]. Godria *et al.* (2025) [12] demonstrated that thyroid dysfunction, with subclinical hypothyroidism being the most common form, was significantly associated with menstrual abnormalities such as oligomenorrhea and menorrhagia. Their study's finding that a considerable portion of reproductive age women with menstrual irregularities had SCH supports our data indicating that SCH contributes to menstrual cycle disruption. Across these prior studies, the underlying mechanism is thought to involve disruption of the hypothalamic pituitary ovarian (HPO) axis by altered thyroid hormone signaling. Thyroid hormones influence gonadotropin releasing hormone (GnRH) secretion and modulate levels of circulating sex steroids, such as estrogen and progesterone, which are crucial for normal menstrual cyclicity. SCH can lead to subtle hormonal imbalances, often increasing prolactin levels and altering luteinizing hormone (LH) and follicle stimulating hormone (FSH) secretion, thereby contributing to menstrual irregularities.

## Conclusion:

In conclusion, comparing our results with previous research highlights consistent evidence that SCH has a meaningful impact on menstrual health in reproductive age women, supporting the importance of thyroid function evaluation in women presenting with menstrual irregularities.

## Figures and Tables

**Table 1:** Demographic characteristics of participants

**Demographic Characteristic**	**SCH Group (n=50)**	**Control Group (n=50)**	**Total (n=100)**
Age (years)	30.5±5.3	29.2±4.7	29.8±5.0
BMI (kg/m^2^)	25.4±4.2	24.7±3.6	25.0±3.9
Menstrual Irregularities	40 (80%)	30 (60%)	70 (70%)
No Menstrual Irregularities	10 (20%)	20 (40%)	30 (30%)

**Table 2:** Thyroid function test results

**Test**	**SCH Group (n=50)**	**Control Group (n=50)**	**p-value**
TSH (mIU/L)	7.2±2.1	2.5±0.6	<0.001
FT4 (ng/dL)	1.2±0.3	1.3±0.2	0.059
FT3 (pg/mL)	3.1±0.6	3.3±0.5	0.098

**Table 3:** Types of menstrual irregularities

**Menstrual Irregularity**	**SCH Group (n=50)**	**Control Group (n=50)**	**p-value**
Irregular Cycle Length	30 (60%)	18 (36%)	0.029
Heavy Menstrual Bleeding	18 (36%)	12 (24%)	0.089
Amenorrhea	2 (4%)	5 (10%)	0.352
Oligomenorrhea	6 (12%)	7 (14%)	0.764

**Table 4:** Comparison of menstrual cycle length

**Group**	**Mean Cycle Length (days)**	**p-value**
SCH Group	33.2±4.7	0.001
Control Group	28.5±3.4	

**Table 5:** Correlation between tsh levels and menstrual irregularities

**TSH Level (mIU/L)**	**Menstrual Irregularity Present (n=70)**	**Menstrual Irregularity Absent (n=30)**	**p-value**
<5.0 mIU/L	20 (28.6%)	18 (60%)	0.002
>5.0 mIU/L	50 (71.4%)	12 (40%)	
